# Jürgen Boeckh (1934–2023) and Vera Boeckh, née von Zwehl (1928–2022): pioneers of sensory physiology and neuroethology

**DOI:** 10.1007/s00359-024-01710-9

**Published:** 2024-06-18

**Authors:** Monika Stengl

**Affiliations:** University of Kassel, Heinrich Plett Str. 40, Kassel, 34132 Germany

**Keywords:** Obituary, German professors, Careers of woman scientists, Postwar period

## Abstract

Jürgen Boeckh, a respected pioneer of insect olfaction died shortly after his beloved wife Vera Boeckh, née von Zwehl, who pioneered insect vision. Both met in 1958, at the Zoological Institute in Munich. There, Jürgen worked in the group of his PhD advisor Dietrich Schneider, while Vera finished her PhD with Werner Jacobs before she joined the group of Hansjochem Autrum. There, Vera characterized the spectral sensitivity of bee photoreceptors, laying the physiological foundation of Karl von Frisch´s behavioral experiments with bee color vision. Meanwhile, Jürgen focused on the physiological characterization of insect antennal olfactory sensilla. In 1962 Vera and Jürgen married in Munich. Sadly, but characteristic of German woman at these times, Vera´s career ended after her marriage, while Jürgen moved with his mentor Schneider to the Max Planck Institute of Behavioral Physiology in Seewiesen near Munich, which became a famous cradle of insect neuroethology. Vera accompanied and supported her husband Jürgen´s career during his scientific *Wanderschaft* which ended in 1969, when Jürgen received a full professorship at the University of Regensburg. There, Jürgen became an accomplished German professor, focusing on insect olfaction from peripheral sensory transduction to information processing in the brain´s antennal lobe. After Jürgens retirement in 2000 they moved to Hopfen, Enzensberg near Füssen, where they enjoyed happy years together, before especially Vera´s health deteriorated. Both died shortly after one another during the Corona pandemic. We lost a remarkable couple of insect scientists that will be remembered as pioneers of sensory physiology and neuroethology.

**Figure 1:**
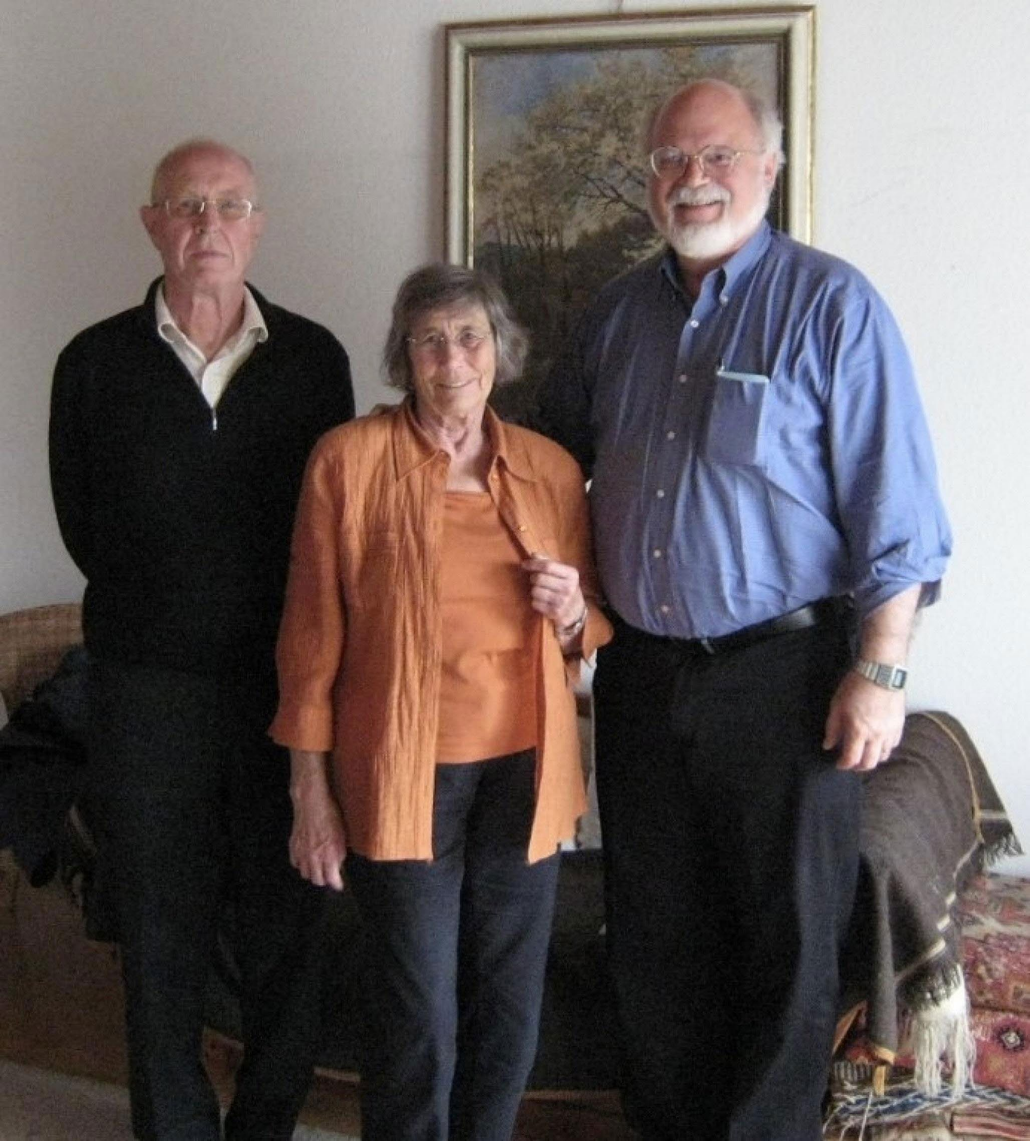
Jürgen and Vera Boeckh with their friend and colleague John G. Hildebrand (photograph by J.G. Hildebrand)

Prof. Dr. Jürgen Boeckh (October 3, 1934 – March 11, 2023) died only three months after his beloved wife Dr. Vera Boeckh (née Dr. Vera von Zwehl, August 14, 1928 – December 13, 2022) had passed away at the retirement home St. Michael in Füssen, Germany. We lost a remarkable couple of scientists who shaped insect sensory neuroscience and were respected representatives of the scientific community in Germany. Both grew up during the oppressive Nazi regime in privileged patriotic conservative upper-class families. During the postwar period, when dual careers were not an option for a couple of married scientists, Vera Boeckh´s career ended early after a brief, but highly successful postdoctoral training. By contrast Jürgen Boeckh became an accomplished German professor, shaping insect chemosensory science.

## Youth and adolescence of Jürgen and Vera during World War II

Jürgen Boeckh was born in Bielefeld 1934, the youngest of four siblings (Peter, Amei, Hans, Jürgen). He grew up in a wealthy upper-class conservative protestant family with his father Dr. med. Rudolf Boeckh descending from a family of powerfully connected protestant priests in Augsburg. Both of his parents were religiously motivated physicians. They served for several years at a protestant mission clinic in Ho-Yuen in China. In 1925, the family returned to Germany, when Jürgen´s father was recruited as physician to the von Bodelschwingh Foundation Bethel in Bielefeld, one of Europe´s largest non-profit welfare and social work organizations of the protestant church that focused on social care of the mentally and physically disabled (Neher 2007). Jürgen´s father believed in the Nazi ideology concerning racial eugenics (*Rassenhygiene*) and supported forced sterilization and euthanasia. However, after the war, following official denazification and exoneration in 1947 (Neher 2007), Rudolf Boeckh became a highly accomplished reformer of psychiatric work focusing on occupational therapy. In 1955, he received the Federal Cross of Merit, first class (*Bundesverdienstkreuz erster Klasse*) for his accomplishments as successful clinic director and for his work as a respected advisor of the Inner Ministery of Interior of Baden-Württemberg for the organization and planning of new clinics (Neher 2007).

Jürgen was born 1934 in Bielefeld, about 10 years after his next brother. When he was two years old his father moved with his family to a new position as chief physician and chief psychiatrist in Neuendettelsau, Bavaria, close to Nuremberg (Neher 2007). There, the protestant church maintained a deaconry-clinic for the mentally and physically disabled, the old and poor that depended on the support of the church. In Neuendettelsau, young Jürgen Boeckh lived with his family in the doctor´s residence directly adjacent to the clinic where both of his parents worked full time as dedicated physicians. As a late born child, Jürgen was often lonely, playing with his huge Saint Bernard dog in the family´s back yard across from the psychiatric clinic. His love for these huge, sweet-natured dogs stayed with him throughout his life, seeking time and again large Newfoundlander dogs as loyal companions.

After his elementary school in Neuendettelsau, during the last year of the war, Jürgen first attended the high school *Domspatzen-Gymnasium* in Regensburg, singing in the famous *Domspatzen*-choir. At that time, both of his parents worked as physicians in the military hospital in Regensburg. It was here, in 1943, that Jürgen´s father became chief physician following several shorter periods of service in military hospitals in the Caucasus and in Romania (Feb. 1941–1942). It is likely that Rudolf Boeckh had moved his family in 1944 from Neuendettelsau to Regensburg, after Jürgen finished elementary school. After the German capitulation in 1945, his father was forced by the victorious American powers to close the military hospital in Regensburg, losing his position as chief military physician (Neher 2007). While his parents struggled with denazification requirements, in search for new positions, Jürgen was sent to the old prestigious protestant boarding school St. Anna in Augsburg, where the powerful protestant family of Rudolf’s father resided. Jürgen remained at St. Anna for almost the complete duration of his adolescence, while his parents rarely had time to visit their youngest child, being busy trying to survive after the end of World War II. These were very lonely and difficult years for Jürgen (personal communication), since at that time Christian education meant maintaining strict discipline, obedience, and order, aiming at high intellectual goals.

Jürgen graduated from high school in 1953. Finally freed from the strict boarding school in Augsburg he moved to the University of Tübingen (Leibnitz-Kolleg, *Studium Generale*, 1953-54) to find out what he wanted to aim for. After realizing that he was not made for medicine, he decided to study zoology. Aiming for a broad education in natural sciences he was determined to learn from distinguished professors at the Universities of Tübingen, Hamburg, and Munich (1954-58). Jürgen´s timing was very fortunate: he found at the Leibnitz-*Kolleg* of Tübingen an excellent research mentor: Dr. Dietrich Schneider, research assistant of Prof. Dr. Alfred Kühn. Schneider was an inspiring, creative scientist and a courageously independent thinker, with the ability to identify and solve the most challenging research questions. Furthermore, Schneider´s humble, supportive personality allowed others to grow next to him, a rare trait amongst German professors of his generation. When Schneider was offered a position by his former PhD advisor, the powerful Prof. Dr. Hansjochem Autrum at the Zoological Institute in Munich in 1958, Jürgen moved with him as his PhD student (Autrum 1996). There, Jürgen met Vera von Zwehl, an intellectually brilliant woman who was also sophisticated, courageous, and independent.

Vera Antonie Caroline von Zwehl was born in Hamburg (August 14, 1928) as the eldest of four siblings. Her father was the chemist and nobleman Dr. Gustav Franz Maria von Zwehl, and her mother Elisabeth, born Pschorr, descended from the wealthy, respected dynasty of the beer brewers Hacker-Pschorr in Munich. During World War II, this very conservative, Catholic, socially esteemed, and wealthy family lived in Naples, Italy, where her father was employed as a chemist. In Naples, Vera von Zwehl attended the Swiss Elementary School (1934–1939) and the high school Ginnassio-Liceo Umberto I (1939–1942). In 1942, when her father obtained a leading position with ICMESA, the Italian branch of the biomedical concern Hoffman-La Roche in Meda close to Milano, her courageous mother moved with her four children to Tegernsee, Bavaria. Thus, Vera grew up during the Nazi regimes in Italy and Germany, where the social value for a woman was tightly connected to a prestigious marriage, devotional motherhood, and being a supportive wife to her husband. After her graduation from high school (*Städtische Oberrealschule*) in Miesbach, Bavaria in 1947, the family reunited with her father Gustav von Zwehl in Meda, Italy (February 1948).

In 1949 young Vera ventured alone for work and travel to Great Britain exploring the world on her own, enjoying freedom and peace. Following her strong intellectual interests and her love for nature, she decided to study biology at the University of Munich (1952–1955). In 1955, she went to the newly founded Max Planck Institute for Behavioral Physiology (Max Planck Institut für Verhaltensphysiologie, MPIV) in Seewiesen, where she took an internship working with Prof. Dr. Konrad Lorenz, the co-founder of ethology and later Nobel laureate in Physiology or Medicine (1973). Konrad Lorenz became the first deputy director under Erich von Holst, and after von Holst´s death he became director of the MPIV in Seewiesen (1961–1973). According to Vera´s own account, her internship in Seewiesen was one of the most intellectually inspiring and happy times for her. It is unclear whether she was aware of Konrad Lorenz´s biology-based Nazi ideology, or of his strong influence on current ethics defining the role of woman in her time (Vicedo 2009). Nevertheless, Lorenz, whom she admired, likely influenced her career choices.

Vera von Zwehl did not manage to secure a position as PhD student to follow her scientific interests in comparative ethology. Instead, in 1960, she finished a comparative neuroanatomical dissertation “System of arteries and veins innervating the brain of teleosts” with Prof. Dr. Werner Jacobs, “Doctor-father” of many renowned scientists. During the last two years of her dissertation, the good looking, bright, and lively Jürgen Boeckh in the lab next door may have come to her attention. After completion of her dissertation with Jacobs, in 1961, Vera von Zwehl worked in the laboratory of Autrum, paid by the German Research Foundation (DFG) as a research assistant (Autrum 1996). Autrum took over the position of Director at the Zoological Institute at the University of Munich after von Frisch retired. Even though Vera was a star during her studies at the University of Munich, being a bright, radiant, and sporty student from an aristocratic family that was idolized by her male colleagues, she now began to encounter envy and the perception of being a competitor for scientific success and prestige. Furthermore, she worked in the lab of Autrum who was convinced that female students cannot successfully combine a career in science with their natural instinct-based womanly goals of marriage and motherhood (Autrum 1996). Nevertheless, in Autrum´s lab Vera had the opportunity to work on a research topic of her interest. Her research project on honeybee vision was based upon comparative behavioral experiments with bees by von Frisch (1919) that she could link to her previous exciting ethological studies with Konrad Lorenz. Furthermore, she could learn a challenging new scientific technique from Autrum´s research assistant Dr. Dietrich Burkhardt (Burkhardt and Autrum 1960) – electrophysiology – which opened new research perspectives.

## Years together and scientific accomplishments

While Dr. Vera von Zwehl worked with Autrum, Jürgen Boeckh, in the lab next door, obtained his doctoral degree (1958–1962) with Dietrich Schneider. Both Jürgen and Vera were working with cutting-edge electrophysiological methods and performed ground-breaking work in the newly emerging scientific field of insect sensory physiology. Jürgen performed extracellular electroantennogram (EAG) and single sensilla-recordings of the sexton beetle, of different moth species, and of cockroaches, analyzing chemoreception (Boeckh 1962; Boeckh et al. 1960, 1963; Schneider and Boeckh 1962). Vera studied insect vision recording from single photoreceptor cells in the compound eye of the honeybee (Autrum and von Zwehl 1962a; Autrum and Zwehl 1962b, 1963, 1964). Her work provided for the first time the physiological basis for trichromatic color vision and for polarized light detection in honeybees that were predicted by previous ground-breaking behavioral experiments of Karl von Frisch.

In the international scientific community Vera´s publications on honeybee photoreception (Autrum and von Zwehl 1962a; Autrum and von Zwehl 1962b; Autrum and von Zwehl, 1964) received even more attention than Dietrich Burkhardt´s innovative publication on flesh fly photoreceptors (Burckhardt and Autrum 1960). However, she was not rewarded adequately for her scientific accomplishments by Autrum. In contrast to her male colleagues, she was denied first authorship for her excellent work. Apparently, she was not perceived by Autrum as an ambitious junior scientist that deserves respect and mentoring. Accordingly, her success with adopting the very challenging electrophysiological method to the eyes of the honeybee was perceived by Autrum as the advantage of the honeybee as model system for electrophysiology rather than due to Vera´s talent and accomplishments (Autrum 1996). Thus, while Jürgen received support, respect, and reward for his scientific ambitions and accomplishments by a generous scientific mentor, Vera was not so lucky.

The year 1962 became a very important milestone for both. Vera von Zwehl and Jürgen Boeckh married October 20, 1962, at the catholic church St. Sylvester in Munich. Jürgen had just finished his dissertation (1962; *Electrophysiologische Untersuchungen an einzelnen Geruchsrezeptoren auf den Antennen des Totengräbers Necrophorus, Coleoptera*) and had obtained a four-year position as research assistant at the MPI for Psychiatry in Munich, where Dietrich Schneider became director first in Munich (1962–1965), then at the MPIV in Seewiesen (1965). Thus, with Dietrich Schneider receiving resources to carry out ground-breaking work on the insect´s sense of smell, junior scientist Jürgen Boeckh prospered in an intellectually stimulating and supportive environment at the MPIV in Seewiesen. However, his wife Vera was not offered an adequate position in science, especially not at an MPI with its male-dominated hierarchical structure. With choosing to marry at this time, especially in conservative, traditional Bavaria, she was expected to be a supportive housewife for her husband who in turn, was expected to focus on his job to earn enough money to support his wife and children. A dual carrier plan in science for both was not an option (Autrum 1996). Gifted with scientific curiosity and intellectual prowess, Vera suffered in her new expected role, and this was not apparent to her male colleagues. But she shared her feelings with trusted female friends.

Meanwhile, during the 1970’s and 1980’s at the MPIV in Seewiesen brilliant scientists shaped the emerging field of neuroethology. In interdisciplinary collaborations, the MPI groups together searched for the neural basis of natural behavior by comparing how different animal species are adapted in physiology and behavior to their respective environmental niches. It was especially challenging to unravel the insect´s sense of smell that coordinated feeding and mating behavior. It proved extremely difficult to determine which of the millions of chemical substances released into the air by plants or animals are behaviorally relevant and thus might be detected by insect chemosensory receptors. The breakthrough came with the identification of the first insect sex pheromones of the silk moth *Bombyx mori* and of the American cockroach *Periplaneta americana* by the biochemists Adolf Butenandt and Martin Jacobson (Butenandt et al. 1959; Jacobson et al. 1963). Pheromones are highly species-specific communication signals with properties of odors and hormones (Karlson and Lüscher 1959). As sex pheromones they orchestrate male/female reproduction, the critical basis for stable speciation. The detection of these species-specific behaviorally relevant odor stimuli paved the way for the study of insect odor detection in Seewiesen. While his mentor Schneider focused on different solitary nocturnal moth species that strongly rely on their sense of smell to locate their dispersed mates in the dark, Jürgen Boeckh used the gregarious nocturnal cockroach *Periplaneta americana* and the desert locust as his model insects (Boeckh et al. 1963, 1965; Boeckh, 1967; Schneider and Boeckh 1962; Schneider 1992).

Together with electrical engineers who constructed equipment needed for cutting edge electrophysiological methods, Schneider and his group recorded the first odor-dependent electrical signals originating in the insect’s antennae, the EAGs. With a recording electrode slid over the cut tip of the insect´s antennal flagellum, and a reference electrode near the base of the antenna contacting the hemolymph, they obtained dose-dependent EAGs after application of pheromone via a refined odor application system (olfactometer). Using these methods, the antennae were identified as the main chemosensory sense organs and the respective species-specific pheromones that the antennae responded to were functionally identified (Schneider 1957; Schneider and Boeckh 1962; Boeckh et al. 1963; for review see: Schneider 1992). As the next step the different types of antennal chemosensory sensilla were characterized physiologically with tip-recordings from single sensory hairs and morphologically with refined neuroanatomical methods (e.g. Steinbrecht 1980). Thus, at the MPIV in Seewiesen Schneider´s group, with Jürgen Boeckh as accomplished member, successfully combined neuroanatomical and electrophysiological studies to characterize the sense of smell in insects for the first time, assigning detection of different odor qualities to specific antennal sensilla types and determining olfactory thresholds and dose-response curves (reviews: Boeckh et al. 1965; Kaissling 1971, 1987; Schneider 1992; Hildebrand 1995; Keil 1997). Interestingly, while pheromone-specific olfactory receptor neurons innervating long trichoid sensilla were highly sensitive and highly specific for only one pheromone component, general odor detecting sensory neurons of shorter hairs were less discriminating and needed higher odor concentrations to respond.

During his time in Seewiesen (1962-66), still having no perspective for long-term employment in science, Jürgen staged well-chosen short international research internships at the prestigious Gatty Marine Laboratory at St. Andrews, Scotland with Adrian Horridge and at the Zoological Station in Naples, Italy. Apparently, Vera followed her husband abroad to these new research locations. Not only with her specific scientific knowledge, but also with her excellence in speaking and writing in Italian and English, she helped her husband Jürgen´s efforts to further qualify for a career in science.

Jürgen Boeckh was aware of the need to develop his own research profile independent of his internationally renowned mentor Dietrich Schneider. In 1966, Jürgen Boeckh accepted a position in Frankfurt. This position was a very timely offer by Prof. Dr. Dietrich Burkhardt, the former PhD student and accomplished scientific coworker of Autrum for many years, who was now full professor at the University of Frankfurt. In these supportive surroundings and employed as a research assistant, Jürgen Boeckh managed the next decisive milestone of his career: his *Habilitation* (1967; *Reaktionsschwelle, Arbeitsbereich und Spezifität eines Geruchsrezeptors auf der Heuschreckenantenne*). From his mentor Dietrich Schneider´s own bad experiences (Autrum 1996), he knew that this career step crucially depends on the good will and support of the respective University faculty. The established professors have the power to decide, not necessarily based solely upon scientific accomplishments, whether an applicant for a *Habilitation* will be allowed to join the society of scientists. Luckily, his *Habilitation* on “Olfactory receptors on the antenna of locusts”, was accepted by the well-meaning faculty at the University in Frankfurt, promoting him to become first assistant, and then, in 1968, associate professor at the University’s Zoological Institute. During the years 1968–1969, while searching still for a full professor position, he spent a sabbatical year at the University of Oregon, USA, with Graham Hoyle and at the Brain Research Center in the laboratory of Konrad Akert at the University of Zurich (Boeckh et al. 1970a). During these years since 1962, Vera Boeckh did not hold any paid position, but supported her husband´s scientific career. The stress of an insecure future, hopping between labs in different cities and different countries, and the looming social expectation that a dedicated scientist is required to fully focus on research and not on private happiness, will not have furthered Vera’s and Jürgen´s wish to become parents.

Finally, based upon his scientific achievements in the fields of neuroethology and insect chemosensation, and his excellent scientific networking at the University of Munich, in 1969, Jürgen Boeckh received a full professor position at the young *Reformuniversität* of Regensburg, Bavaria. It was certainly helpful that Hansjochem Autrum was head of the advisory board which provided advise for the ministry of education that founded the University of Regensburg and several other German universities in short temporal sequence (Autrum 1996). Furthermore, at the *Reformuniversität* of Regensburg it was possible for the first time to personally apply for a full professorship. Until then, young scientists had to wait for being chosen and appointed for full professorships (*einen Ruf erhalten*) by established German professors. When at the newly founded University of Regensburg two open positions for department heads in zoology were advertised without the typically narrow denomination, Jürgen Boeckh teamed up with his colleague Dr. Helmut Altner (Altner and Boeckh 1967; Boeckh and Altner 1977) from Munich, a former student of Autrum (Autrum 1996). In a novel joint application with an attractive synergistic research concept, both applied for both open positions. First, Helmut Altner was appointed in 1968, while Jürgen Boeckh had to wait until Franz Huber, the first on the other appointment list before Jürgen, declined the offer to move from his position at the University of Cologne to the University of Regensburg. Finally, when a third full professorship in zoology was advertised, Prof. Dr. Dietrich Burkhardt (Univ. of Frankfurt) was applying successfully to move from his full professorship in Frankfurt to Regensburg.

With a trifecta of department heads of zoology at the University in Regensburg: Prof. Dr. Helmut Altner became head of the Department of Zoology I in 1968 (functional morphology of sense organs, focus thermo- and hygroreceptors), in 1969, Prof. Dr. Jürgen Boeckh became head of the Department of Zoology II (insect olfaction), and Prof. Dr. Dietrich Burkhardt head of the Department of Zoology III (insect vision), likely in 1970. Since all three of them focused their research on the timely synergistic research topic of insect physiology/neuroethology, in 1979, on the initiative of their DFG referent Mrs. Dr. John with Jürgen Boeckh as head/speaker, they successfully applied for DFG-funding for the first largest DFG program (1979–1993): structure function adaptations in sensory systems (*Sonderforschungsbereich =* SFB #4 *Sinnesleistungen: Anpassung von Strukturen und Mechanismen*). This prestigious DFG-program secured financial support for the research at all three zoology departments at the University of Regensburg for 15 years and was expertly managed by academic director Dr. Peter Streck. All three department heads had been supported by their partners/wives, each of whom were also bright and excellently trained PhD scientists. Sadly, all three department heads failed to negotiate scientific positions for their partners. Due to anti-nepotism regulations, the head of the University advised the wives of Altner and Boeckh to stop working in their husbands´ laboratories. Only Dr. Ingrid de la Motte who did not yet marry her partner Dietrich Burkhardt, continued to work together with him on the visual system of the fascinating stalk-eyed flies (Burkhardt and de la Motte 1972; Burkhardt and Motte 1983).

Only two joint publications documented Vera Boeckh´s work with her husband (Ernst et al. 1977; Boeckh and Boeckh 1979). It must have been very frustrating for these intelligent woman scientists (Dr. Elisabeth Hansen-Delkeskamp, the wife of Prof. Dr. Kai Hansen at the Department of Zoology, also being one of them). They were not only denied employment, but also denied respect as fully-fledged scientists, and now their memories slowly fade away from science. Furthermore, none of these scientific couples had children. Nevertheless, lively, practical Vera, did not become bitter or frustrated after having to give up experimental science, but instead worked as a translator of scientific textbooks. Furthermore, because private funds from her wealthy family allowed it, she enjoyed frequent travel, either alone or with her husband, to Nepal, Africa, and the far East. Driven by her broad intellectual interests, she joined for a time the community of the Tuaregs, nomads in the Sahara of northern Africa, studying their culture, as well as happily surveying African fauna and flora. Both Vera and Jürgen Boeckh were passionate alpinists and skilled skiers. Both loved classical music, arts, and crafts, with a special love for beautiful Kelims that lined the walls of their home. Their large Newfoundlander dogs were their consistent companions.

During his time in Regensburg, well-funded by the DFG´s SFB 4, Jürgen Boeckh could develop long-term research projects with different insect species for the first time, venturing from the sensory periphery into the brain´s antennal lobes, the projection areas of the antennal sensory neurons (Ernst et al. 1977; Boeckh et al. 1970a, 1984). With refined neuroanatomical techniques, such as electron microscopy and single cell labeling during different times of development, his lab deciphered the connectivity of cell types in the cockroach antennal lobe and showed that convergence of sensory neurons improved the signal-to-noise ratio of postsynaptic projection neurons (Malun et al. 1993; Salecker and Boeckh 1996; Distler and Boeckh 1996, 1998; Boeckh et al., 1970). In addition, his lab demonstrated for the first time that the long cockroach antennae house the local receptive fields of pheromone-sensitive neurons (Hösl 1990). In contrast to his earlier studies, Jürgen now emphasized state-of-the-art neuroanatomical studies and focused less on functional studies (Burrows et al. 1982). Later, he also added blood sucking insects such as mosquitos, which are important disease vectors, to his model insects and included behavioral studies in his lab´s experimental repertoire (Pappenberger et al. 1996; Geier et al. 1999; Steib et al. 2001).

As university professor, Prof. Dr. Jürgen Boeckh now had many other obligations and opportunities which motivated him to get involved in science politics. He became dean of the Faculty of Biology and Preclinical Medicine at the University of Regensburg (1970–1971; 1983–1985), was kept very busy as speaker of the SFB 4 (1979–1993), became president of the German Zoological Society (1981–1983), was executive editor of “Chemical Senses”, and in 1988, became principal advisor and referee for zoology of the DFG. These many important administrative positions, especially as principal referee for all DFG grant applications in zoology in Germany, guaranteed considerable political power and very high responsibility in the German research community. These important duties occupied much of his time together with a heavy load of administration and teaching obligatory for each German University professor. Jürgen Boeckh was fortunate to have a permanent professional staff such as academic director Dr. Peter Streck that reliably organized and performed the ample administrative and technical work required to maintain a successful research laboratory. Next to administrative tasks, his staff taught the required courses in zoology and provided technical continuity and excellence in sophisticated methods such as electrophysiology and neuroanatomy/electron microscopy to new generations of students in his lab. During continuous funding of his SFB 4, nine of the SFB´s principal investigators received permanent professorships, four received the prestigious Heisenberg-Stipends of the DFG, and nine SFB young investigators successfully completed their habilitation. Furthermore, his lab´s new members Dr. Martin Geier and Dr. Andreas Rose founded a spin-off company (Biogents AG) based on their scientific work with blood-sucking insects.

Jürgen Boeckh remained in Regensburg, despite having been offered permanent positions at the universities of Ulm (1974) and Munich (1978). In 2000, he retired. While Vera and Jürgen Boeckh had lived together mostly in Schönhofen near Regensburg (1969–2000), after Jürgen´s retirement they moved to Hopfen, Enzensberg, near Füssen (2000–2022). After cumulative physical and mental deterioration, Vera became seriously ill. Jürgen cared for her in their own house, before he moved with her to a nearby retirement home, where Jürgen´s father had also stayed until his death. Shortly after their move Vera died, breaking Jürgen´s heart. Only three months after Vera, after a violent pneumonia following an infection with Covid 19 Jürgen died unexpectedly in the clinic St. Vinzenz in Pfronten.

With the deaths of Prof. Dr. Jürgen Boeckh and Dr. Vera Boeckh, we lost a remarkable pair of scientists and impressive personalities that we will remember with great respect (see also obituary by Steinbrecht et al. 2023; *Mitteilungen der Dt. Zoologischen Gesellschaft*).

## Data Availability

No datasets were generated or analysed during the current study.
